# Therapeutic Strategies Against Cancer Stem Cells in Esophageal Carcinomas

**DOI:** 10.3389/fonc.2020.598957

**Published:** 2021-02-16

**Authors:** Plabon Kumar Das, Farhadul Islam, Robert A. Smith, Alfred K. Lam

**Affiliations:** ^1^Department of Biochemistry and Molecular Biology, University of Rajshahi, Rajshahi, Bangladesh; ^2^Institute for Glycomics, Griffith University, Gold Coast, QLD, Australia; ^3^Centre for Genomics and Personalised Health, Genomics Research Centre, School of Biomedical Sciences, Institute of Health and Biomedical Innovation, Queensland University of Technology (QUT), Kelvin Grove, QLD, Australia; ^4^Cancer Molecular Pathology, School of Medicine, Griffith University, Gold Coast, QLD, Australia; ^5^Faculty of Medicine, The University of Queensland, Brisbane, QLD, Australia

**Keywords:** esophageal cancer, esophageal cancer stem cells, cancer signaling, miRNAs, hypoxia, autophagy, therapeutic options

## Abstract

Cancer stem cells (CSCs) in esophageal cancer have a key role in tumor initiation, progression and therapy resistance. Novel therapeutic strategies to target CSCs are being tested, however, more in-depth research is necessary. Eradication of CSCs can result in successful therapeutic approaches against esophageal cancer. Recent evidence suggests that targeting signaling pathways, miRNA expression profiles and other properties of CSCs are important strategies for cancer therapy. Wnt/β-catenin, Notch, Hedgehog, Hippo and other pathways play crucial roles in proliferation, differentiation, and self-renewal of stem cells as well as of CSCs. All of these pathways have been implicated in the regulation of esophageal CSCs and are potential therapeutic targets. Interference with these pathways or their components using small molecules could have therapeutic benefits. Similarly, miRNAs are able to regulate gene expression in esophageal CSCs, so targeting self-renewal pathways with miRNA could be utilized to as a potential therapeutic option. Moreover, hypoxia plays critical roles in esophageal cancer metabolism, stem cell proliferation, maintaining aggressiveness and in regulating the metastatic potential of cancer cells, therefore, targeting hypoxia factors could also provide effective therapeutic modalities against esophageal CSCs. To conclude, additional study of CSCs in esophageal carcinoma could open promising therapeutic options in esophageal carcinomas by targeting hyper-activated signaling pathways, manipulating miRNA expression and hypoxia mechanisms in esophageal CSCs.

## Introduction

Esophageal cancer (EC) is the seventh most common malignancy around the world and the sixth most leading cause of cancer-related mortalities with an estimated 572,000 new incidences and 509,000 deaths in 2018 (1, 2). There are two histopathological subtypes of esophageal cancer such as esophageal squamous cell carcinoma (OSCC) and esophageal adenocarcinoma (OAC) (3–5). The incidence of OAC has been escalating in the Western world, whereas OSCC is more common in the Asia-Pacific region (1). Currently, patients with either subtype receive similar treatment, which is a neoadjuvant chemo-radiotherapy followed by surgery (5). The clinical outcome of the standard therapeutic regimen is, however, limited, as much as 20% of tumors do not respond to chemo-radiotherapy at all, and more than 50% do not respond sufficiently. Furthermore, even after complete responses to adjuvant therapy, early and distant relapse occurs in most cases (5). Therefore, in-depth research is required to investigate the underlying mechanisms of therapy resistance and the subpopulation of cancer cells causing therapy failure needs to be thoroughly investigated.

Accumulating information from research has revealed that a subpopulation of cancer cells known as cancer stem cells (CSCs) are associated with clinical features such as drug resistance, self-renewal, and tumorigenicity in esophageal cancer (6–10). Several pathways *e.g.* Wnt/beta-catenin, Hedgehog, Notch, JAK-STAT3 and Hippo pathways are hyper-activated in both OSCC and OAC, especially in esophageal CSCs. These pathways drive proliferation, differentiation, stemness, and resistance to therapy in the tumors in which they are activated (11–16). For example, the Wnt/beta-catenin pathway was found to contribute to CSC renewal, whereas the Hedgehog pathway has been found to play profound roles in regulating proliferation, not only of normal embryonic cells, but also of cancer cells (11, 13). In addition, altered expression of micro-RNAs; tumor microenvironmental factors such as autophagy, and hypoxia; and reactivation of epithelial-mesenchymal transition (EMT) alone or in combination can trigger the pool of CSCs by aberrant activation of signaling pathways, resulting in the development of cancer recurrences and treatment resistance in esophageal cancer (17–19).Therefore, further investigation regarding the function of CSCs or their associated pathways could provide new potential therapeutic options against esophageal cancers.

Novel therapeutics targeting CSCs rather than bulk-cancer cells or later differentiated progenitors could provide many benefits in patients with esophageal cancer. Traditional cytotoxic agents cannot target CSCs properly as a majority of anti-tumor drugs at present are DNA damage inducing agents (20). They induce tumor cell death most effectively during cell division, while CSCs are usually dormant and do not enter the cell cycle. Thus, DNA damaging agents have little capacity to not induce the death of CSCs (20). Moreover, several mechanisms have been identified in CSCs to avoid DNA damage-induced cell death. For example, CSCs enhance ROS scavenging to inhibit oxidative DNA damage, promote DNA repair capability through ATM and CHK1/CHK2phosphorylation, and activate anti-apoptotic signaling pathways, such as PI3K/Akt, WNT/b-catenin, and Notch signaling pathways to escape DNA damaging agent mediated insults (21).

Interestingly, several therapies that specifically target CSCs or their components in the tumor microenvironment are making their way into clinics. Thus, in this review, we undertake a comprehensive overview of the literature regarding the role of CSCs in esophageal cancer. Moreover, the review also discusses potential therapies targeting aberrantly activated signaling pathways, miRNA expression and hypoxia regulated signaling in esophageal CSCs.

## The Role of Cancer Stem Cells in Esophageal Cancer

Cancer stem cells (CSCs) harbor unique properties, such as self-renewal, tumor maintenance (proliferation), invasion and migration, immune evasion, and therapy resistance (22, 23). Virchow and Conheim first proposed that CSCs exist as a subpopulation of cancer cells, which possess the traits of embryonic cells, including the ability to proliferate different lineages and renew themselves (24). They further assumed that cancer is derived from dormant stem-like cells of the same tissue (24). An experimental approach using leukemia stem cells provided the first evidence of the existence of a cell population having the capacity to initiate a secondary tumor, confirming the presence of CSCs (25). In general, there are two hypotheses that have been proposed regarding the origin of CSCs (5). Firstly, normal stem cells can be transformed into CSCs because of genetic and epigenetic alterations. Secondly, dedifferentiated cancer cells acquire the capabilities of CSCs by the process called cellular plasticity (22, 23, 25–27). CSCs often display resistance to therapy, the exact mechanisms of which are not clear, however, a number of underlying mechanisms have been identified *i.e.* enhanced DNA repair efficiency, increased expression of detoxification enzymes (ALDH), increased expression of drug resistance proteins, up-regulation of anti-apoptotic proteins (Bcl-2, Bcl-xL, Mcl-l, Bcl-w), mutations in key signaling molecules, and overexpression of drug efflux pumps (P glycoprotein 1, ABCG2) etc. in CSCs (28, 29).

Esophageal CSCs directly regulate cancer initiation, progression, metastasis, therapy resistance and recurrence both in esophageal adenocarcinomas (OAC) and esophageal squamous cell carcinomas (OSCC) (26, 30, 31). CSCs of esophageal cancer can be identified and isolated by specific cell surface and intracellular markers. For example, cell surface and intracellular markers such as CD44, ALDH, Pygo2, MAML1, Twist1, Musashi1, CD271, and CD90, are used to identify CSCs, whereas, stem cell markers including ALDH1, HIWI, Oct3/4, ABCG2, SOX2, SALL4, BMI-1, NANOG, CD133, and podoplanin were associated with the enrichment of CSCs in OSCC (26, 30, 31). In addition, isolation of side population (SP), a subpopulation of cells with the ability to exclude dyes such as Hoechst 33342, are enriched with stem cells and SP isolation can be used to identify CSCs in OSCC (31). According to several studies, side population has been utilized in the isolation of CSCs from esophageal cancer (32–34). For example, isolation of side population in different esophageal cancer cells such as OSCC (OE21) and OAC (OE19, OE33, PT1590, and LN1590) revealed that the proportions of side population cells are varied among the cell lines and they are resistant to chemotherapy (34). Also, SP cells exhibited stem-like cell phenomena such as epithelial-mesenchymal transition (EMT) (34). The stem-like esophageal cells also become more radio-resistant than parental cells (35). The radio-resistant property of esophageal CSCs is attributed to the overexpression of β-catenin, Oct3/4, and β1-integrin (36). Moreover, esophageal CSCs dictate intrinsic and acquired chemotherapy resistance to 5-fluorouracil (5-FU) and cisplatin in OAC (22). This therapy resistance is associated with changes in the regulation of EMT (22). Additionally, recent studies demonstrated a relationship between the expression of miRNAs, for example, miR-296 (37) and miR-200c (38) and chemoresistance in esophageal CSCs. Furthermore, overexpression of *WNT10A*, a member of the *Wnt* gene family, increases self-renewal capabilities of CSCs and induces a larger population of CSCs in OSCC (39). Most importantly, CSCs with increased tumorigenicity were formed when tumors multiply and experience treatment threats such as targeted agents, cytotoxic agents or radiation (19). Therefore, it is plausible that eradication of CSCs or, alternatively, reduction of their malignant and stemness properties can result in more successful therapeutic approaches.

## Targeting Signaling Pathways in Esophageal Cancer Stem Cells

The signaling pathways which trigger embryogenesis also play a significant role in oncogenesis (40). The pathways highly associated with the maintenance of esophageal CSCs include Wnt/β-catenin, Notch, Hh, and Hippo pathways (39). These pathways are involved in maintaining tissue homeostasis and normal stem cell renewal and dysregulation of these signaling pathways drives esophageal CSCs formation (39). For example, a Wnt/β-catenin activator WNT10A is highly expressed in OSCC tissue. Consistently cells with the expression of WNT10A showed enrichment for CD44+/CD24−, and these cells showed increased self-renewal, invasive and metastatic potential (40, 41). Notch signaling is another prominent driver of cancer stemness in OAC. Experimental work shows, for example, that inhibiting Notch pathway by γ-secretase inhibitors reduces the size of patient-derived xenograft tumors of OAC in mice (42). Furthermore, aberrant activation of these pathways can result from autophagy, hypoxia, anti-cancer therapy and EMT, alone or in combination with each other, which subsequently leads to an enrichment of CSCs and development of recurrences, metastasis and increasing treatment resistance (39). These phenomena can be manipulated by novel therapeutics targeting specific components involving the stemness of cancer cells to offset their role in treatment resistance.

## Targeting the Wnt/β-Catenin Pathway in Esophageal Cancer Stem Cells

**Figure 1:**
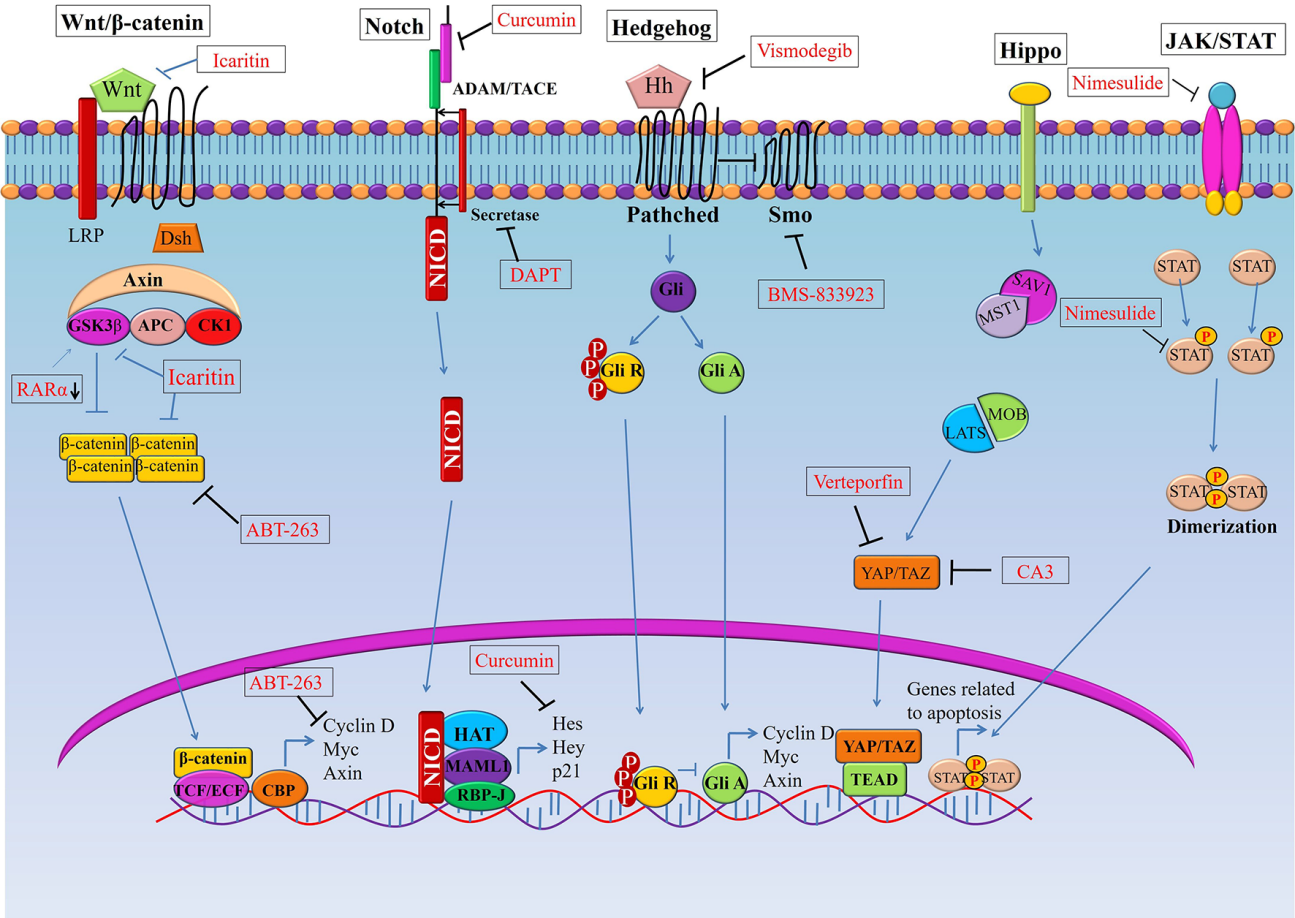
Targeting signaling pathways in esophageal cancer stem cells (CSCs). Schematic representation of the Wnt, Notch, Hedgehog (Hh), and Hippo pathways in esophageal CSCs. Novel therapeutics (synthetic and natural) kill CSCs by targeting these signalling pathways or their components.GSK3β, Glycogen synthase kinase 3 beta; Dsh, Disheveled; APC, Adenomatous polyposis coli; CK1, Casein kinase 1; TCF, T-cell factor/lymphoid enhancer factor; SMO, Smoothened; YAP, (Yes-associated protein); TAZ, Transcriptional coactivator with PDZ-binding motif.

The Wnt/β-catenin signaling pathway plays a pivotal role in oncogenesis through different mechanisms (43). In normal physiological conditions, the Wnt/β-catenin pathway controls the expression of downstream genes, which are involved in basic cellular and biological functions including proliferation, differentiation, apoptosis, and cell death (44). Thus, in order to exert normal physiological functions, activation of Wnt/β-catenin signaling should be kept at the normal level. However, aberrant activation of this pathway is associated with many cancers including esophageal cancer. For instance, over-activation of the Wnt/β-catenin pathway can be an underlying factor of progression, metastasis, and invasion in OSCC by inducing a CSC phenotype (40). Therefore, targeting the Wnt/β-catenin pathway has potential for the inhibition of CSC growth. Though Wnt/β-catenin inhibitors are in clinical trials for various solid tumors, inhibitors are yet to reach clinical trials in esophageal cancer (39). Emerging molecules inhibiting Wnt/β-catenin signaling have provided promising preclinical outcome against esophageal cancer (**Figure 1**, **Table 1**). For example, Icaritin, an alkaloid extracted from *Herba epimedii*, was found to reduce the growth of CSCs derived from the OSCC cell line ECA109 by inhibiting Wnt/β-catenin and Hedgehog pathway (45). Icaritin inhibited proliferation, migration, and invasion of CD133+ esophageal CSCs in a dose-dependent manner and enhanced the apoptosis of these stem cells. In addition, Icaritin induced up-regulation of GSK3β and down-regulation of Wnt and β-catenin, Hedgehog, Smo, and Gli proteins in Wnt/β-catenin and Hedgehog pathways, respectively (45).

**Table 1:** Targeting signaling pathways in esophageal cancer stem cells.

Compounds/Drugs/Process	Carcinomas	Target Pathways	Functions	Reference
Icaritin	OSCC	Wnt/β-catenin	Inhibits the proliferation, migration, and invasion of CD133+ CSCs by up-regulating GSK3β and down-regulating Wnt and β-catenin proteins	(45)
ABT-263	OAC and OSCC	Wnt/β-catenin	Reduces the expression of β-catenin protein level, which subsequently results in downregulation of its target protein cyclinD1 in both OAC and OSCC Selectively kills ABCG+ CSCs and inhibits tumor sphere formation in both OAC and OSCC Reduces tumor volume and tumor weight alone or in combination with 5-flurouracil in both OAC and OSCC	(46)
Retinoic acid receptor α (RARα) knockdown	OSCC	Wnt/β-catenin	RARα knockdown inhibits the proliferation and metastasis of OSCC cells by minimizing the expression of PCNA, Ki-67, MMP7, and MMP9 It also enhances drug susceptibility of OSCC cells to 5-fuorouracil and cisplatin	(47)
*N-*[*N-(3, 5-difluorophenacetyl-L-alanyl)*]*-S-phenylglycine t-butyl ester (*DAPT)	OAC	Notch	Reduces Notch-mediated transcription and subsequently decreases transcription of Notch target genes Decreases cell viability, the number and size of colony formation	(42)
Blockade of Gene amplified in squamous cell carcinoma 1 (GASC1)	OSCC	Notch	Blockade of GASC1 results in inhibition of OSCC stemness property Reduces the expression of Notch1	(48)
Vismodegib	OAC	Hedgehog	Blocks the interaction between the Ptch-receptors and their ligands Reduces CSC pool in OAC	(49)
Silencing of ATPase family AAA domain containing protein 2 (ATAD2)	OSCC	Hedgehog	Silencing of ATAD2 or inhibiting the Hedgehog signaling decreased the proliferation, invasion and migration abilities along with colony formation of CSCs in OSCC	(50)
CA3	OAC	Hippo	Inhibits proliferation, induces apoptosis, reduces tumor sphere formation of ALDH1+ cells	(35)
Metformin	OAC and OSCCC	mTOR	Decreases the expression of stem cell signaling markers such as *Jagged1*, *Shh*, *YAP1* in both OAC and OSCC Effectively downregulates mTOR components including phospho-AKT, phospho-S6, phospho-70S6 in both OAC and OSCC Inhibits the growth of carcinoma cells *in vitro* and *in vivo* in both OAC and OSCC	(51)
Nimesulide	OSCC	JAK/STAT	Inhibits Cyclooxygenase-2 expression which subsequently diminishes JAK/STAT signaling leading to the suppression of OSCC cell growth and increase of apoptosis	(52)
Erlotinib and Cetuximab	OSCC	EGFR	Halts EMT by instigating differentiation in non-CSC populations	(53)
Pristimerin	OSCC	NF-kβ	Suppresses tumor necrosis factor α (TNFα)‐induced Iκ Bα phosphorylation, p65 translocation, and the expression of NF‐κB‐ dependent genes expression Inhibits proliferation, migration, invasion of OSCC cells and induces apoptosis, and eliminates CSCs like cells	(54)

OSCC, esophageal squamous cell carcinoma; OAC, esophageal adenocarcinoma; CSC, cancer stem cell.

ABT-263, a potent Bcl-2 family inhibitor inhibits cell proliferation and induces apoptosis of human esophageal cells, especially CSCs derived from OAC cell lines (FLO-1, SKGT-4, BE3 and OE33) and OSCC cell lines (YES-6 and KATO-TN) (46, 55). ABT263 reduces the expression of many oncogenes, including genes associated with stemness pathways such as Wnt and YAP/SOX9 axes. Treatment of esophageal CSCs with ABT-263 alone and in combination with 5-FUresulted in the reduction of β-catenin and its target cyclinD1, as well asYAP-1 and its target SOX9 in a dose-dependent manner (46). In addition, ABT-263 selectively kills ABCG+ CSCs and inhibits tumor sphere formation of esophageal CSCs (both OSCC and OAC). Also, ABT-263 alone or in combination with 5-fluorouracil reduced tumor volume and tumor weight in a xenograft model. These treatments dramatically reduced the level of YAP1, SOX9 and the proliferation marker Ki-67 in xenotransplanted tumors of both OSCC and OAC cells (46).

Retinoic acids play a crucial role in embryogenesis, differentiation, and tumorigenesis, which are controlled by retinoic acid receptors (RARs) and retinoid X receptors (RXRs) (56). RARα knockdown suppresses the proliferation and metastasis of OSCC cells by minimizing the expression of proliferative markers (PCNA, Ki-67) and matrix metallo-proteinases (MMP7 and MMP9) (47). Not only that, RARα knockdown also enhances drug susceptibility of OSCC cells to 5-fluorouracil and cisplatin (47). On top of that, RARα knockdown results in inhibition of Wnt/β-catenin pathway by decreasing GSK3βphosphorylation at Ser-9 and inducing phosphorylation at Tyr-216, which subsequently results in reduced expression of its downstream targets such as MMP7, MMP9, and P-glycoprotein. Therefore, targeting Wnt/β-catenin or their components to inhibit the pathway should be effective to halt the growth of CSCs in OSCC (47). Moreover, a few Wnt inhibitors such as PRI-724, LGK-974, Vantictumab and OMP-54F28 are in clinical trials as a single agent or in combination with conventional therapy for many solid cancers (57).

## Targeting Notch Signaling in Esophageal Cancer Stem Cells

Notch signaling is highly activated in less differentiated tumors and drives CSC phenotypes and carcinogenesis in both OSCC and OAC (39, 42). This signaling helps to maintain a robust population of CSCs, thereby resulting in therapy resistance and cancer recurrence (38, 40). Notch inhibition depletes CSC populations in tumors and sensitizes cancer cells to chemotherapeutic agents, which leads to promising response toward neoadjuvant chemotherapy (NAC) in patients with both OSCC and OAC (**Figure 1**, **Table 1**). For example, blocking Notch pathway by DAPT (N-[N-(3, 5-difluorophenacetyl-L-alanyl)]-S-phenylglycine t-butyl ester), a commonly used gamma-secretase inhibitors (GSI), is effective in downsizing tumor growth of OAC. Efficacy of the treatment was shown by a dramatic reduction of the intracellular domain of the notch protein (NICD) in esophageal adenocarcinoma cells (OE33). There was also a reduction in Notch-mediated transcription and a subsequent decrease in the transcription of Notch target genes (42). Treatment of OAC cells with DAPT caused a decrease in cell viability, as well as reducing the number and size of colonies formed by OAC (OE33 and JH-EsoAd1) cells. The inhibition of the Notch pathway caused a significant reduction in transcription of several stem cell marker genes, including *ALDH*, *CD24*, *LGR5*, *SOX2* and *TWIST1*. Furthermore, patient-derived xenograft models clearly demonstrated that inhibition of Notch signaling by gamma-secretase inhibitors is efficacious in downsizing tumor growth (42). Thus, inhibition of Notch signaling by DAPT could impair the stemness of OAC cells *i.e.* esophageal CSCs, resulting in reduced tumor growth in both *in vitro* and *in vivo*.

Gene amplified in squamous cell carcinoma 1 (GASC1), plays a critical role in maintaining self-renewal and differentiation potential of embryonic stem cells (48). GASC1 epigenetically controls the stemness of OSCC by regulation of Notch1. Examination of the expression of GASC1 in OSCC cells and tissues indicated that GASC1 expression is increased in poorly differentiated OSCC (48). Consistent with this observation, patients with OSCCs expressing GASC1 presented a significantly worse survival rate than those without. Most importantly, GASC1 expression in purified CSCs (ALDH+) cells was higher than that in non-CSCs (ALDH−) cells. Several stemness phenotypes of CSCs from OSCC were dramatically decreased after GASC1 blockade, which subsequently resulted in reduced Notch1 expression *via* demethylation of Notch1 promoters (H3K9me2 and H3K9me3). However, the impaired stemness property of CSCs from OSCC followed by GASC1 inhibition was reversed with exogenous Notch1 overexpression (48). This finding suggested that GASC1 promoted stemness in OSCC CSCs cells *via* Notch1 promoter demethylation (48). Therefore, the GASC1/Notch1 signaling axis could be a potential therapeutic target against CSCs of OSCC.

## Targeting Hedgehog Signaling in Cancer Stem Cells of Esophageal Cancer

The Hedgehog (HH) signaling pathway plays a crucial role in growth and differentiation during embryonic development (58). However, abnormal activation of this pathway may also lead to cancer stemness along with stimulation of EMT, cancer metastasis and therapy resistance (59–61). Furthermore, activation of the Hedgehog pathway associated with distant metastases, advanced tumor stage in patients with esophageal cancers (both OSCC and OAC) (60, 62, 63). Although Hedgehog inhibitors have been extensively studied in clinical trials for different solid tumors, clinical trials on esophageal cancers are still limited (64). Vismodegib, also known as GDC-0449, is a small molecule inhibitor of Hedgehog signaling that blocks the interaction between the Ptch-receptors and their ligands (**Figure 1**, **Table 1**) **(**65). In addition, Vismodegib in combination with chemotherapy (FOLFOX) did not increase the survival of patients with gastroesophageal junction adenocarcinoma significantly (64).

Importantly, Vismodegib combined with neoadjuvant chemo-radiotherapy is under investigation in a clinical trial in Hedgehog activated OAC cells (49). Vismodegib treatment reduced the CSC pool derived in OSCC (OE21) and OAC (OE33) cells. Investigation of options for the suppression of the Hedgehog pathway may have additional importance, it has been suggested that neoadjuvant chemo-radiotherapy may activate the Hedgehog pathway, which in turn causes acquisition of more CSC features including the property of therapy resistance (49). For example, there is a subset of cancer cells with activated Hedgehog pathway prior to therapy that renders them able to survive chemo- and radiotherapy (66–69). By contrast, inhibiting the Hedgehog pathway resulted in a reduction of cells with CSC phenotype (CD44+/CD24−), inhibited sphere-forming capability and induced radio-sensitivity (70–72).

BMS-833923, an inhibitor of smoothened (SMO), another constituent of the Hedgehog pathway, combined with chemotherapy (FOLFOX) is currently under investigation in patients with metastatic esophageal carcinoma (73). SMO brings about the translocation of Gli protein into nucleus which results in the transcription of downstream target genes. Other SMO inhibitors such as Sonidegib and Taladegib are being explored currently against gastroesophageal adenocarcinomas (73, 74). In addition, activation of Hedgehog signaling could be inhibited by targeting transcription factor ATPase family AAA domain-containing protein 2 (ATAD2) (73). ATAD2, a member of the AAA + ATPase family, which is involved in various cancers by regulating cell proliferation, apoptosis, invasion and migration, and its overexpression is associated with poor prognosis of patients with cervical and gastric cancer (75, 76). High expression of ATAD2 has been identified in various types of tumors, including OSCC (75, 77). Interestingly, inhibition of ATAD2 resulted in subsequent inhibition of the Hedgehog signaling pathway, which was confirmed by reduced expression of Gli1, SMO, and Ptch11 in OSCC (50). On top of that, silencing of ATAD2 or inhibiting the Hedgehog signaling decreased the proliferation, invasion and migration abilities along with colony formation of CSCs in OSCC. Furthermore, increased apoptosis followed by the suppression of Hedgehog signaling was noted in CSCs derived from OSCC cells (50). Moreover, *in vivo* experiments in nude mice further validated the suppressive effect of siRNA mediated ATAD2 silencing on tumor growth (50). Thus, down-regulation of ATAD2 can certainly restrict the malignant phenotypes of OSCC cells through inhibition of the Hedgehog signaling pathway in CSCs derived from OSCC cells. These findings suggest that targeting the Hedgehog pathway *via* any of a number of mechanisms could be an effective approach to control CSCs in esophageal carcinomas.

## Targeting Hippo Signaling of Esophageal Cells of Esophageal Cancer

The Hippo pathway has been implicated in the regulation of organ size, proliferation, and stem cell properties (78, 79). YAP1 plays a significant role in the maintenance of stemness of embryonic stem cells as well as contributing to the functions of CSCs (80–82).Therefore, deregulation of Hippo and activation of YAP1 in CSCs contributes many important properties of tumors, and thus, targeting YAP1 will be an effective strategy to target CSCs, thereby inhibiting tumor growth.

Several small-molecule inhibitors have been tested against the Hippo pathway in both OSCC and OAC cells (**Figure 1**, **Table 1**) (35, 80, 83–86). For example, a novel YAP inhibitor CA3 exhibited remarkable inhibitory activity on the transcriptional activity of YAP1/transcriptional enhanced associate domains (TEAD) (35). CA3 demonstrated strong inhibitory effects on the growth of OAC, especially on YAP1 overexpressing cancer cells both *in vitro* and *in vivo* (35). Most importantly, radio-resistant CSCs with aggressive phenotypes can be effectively suppressed by CA3 treatment. CA3inhibited proliferation, induced apoptosis and reduced tumor sphere formation of CSC (ALDH1+) cells derived from OSCC (35). Furthermore, CA3 in combination with 5-FU inhibited the growth of esophageal adenocarcinoma, especially in YAP1 overexpressing cancer cells (35). Taken together, these findings suggested that CA3 represents a new inhibitor of YAP1 and primarily targets YAP1 overexpressing and therapy-resistant CSCs generated from OAC.

Additionally, YAP1activity correlated with SOX9 expression in esophageal adenocarcinoma (35). SOX9 was found to be highly upregulated in various premalignant lesions and in tumor tissues and plays crucial roles in tumor development (83–85). The co-activator of Hippo pathway (YAP1) acts as a major determinant of CSC properties in non-transformed cells and as well as in OAC cells which directly upregulates the expression SOX9 (80). YAP1 regulates the transcription of SOX9 through a conserved TEAD binding site in the SOX9 promoter region. Exogenous expression of YAP1 or inhibition of its upstream negative regulators *in vivo* caused an increased SOX9 expression, which subsequently results in the acquisition of CSCs properties (80). On the other hand, shRNA-mediated knockdown of YAP1 or SOX9 in transformed cells inhibited CSC phenotypes *in vitro* and tumorigenicity *in vivo* (80). Furthermore, Verteporfin (VP), a small-molecule inhibitor of YAP1, significantly blocks CSCs (ALDH+ cells) properties in OAC cells overexpressingYAP1 (80). Thus, in the acquisition of CSC propertiesYAP1 driven SOX9 expression is critical, indicating that YAP1 inhibition might be an attractive option in targeting CSC population in esophageal cancer. For example, overexpression of YAP1 was positively associated with CDK6expression in radiation-resistant esophageal cancer tissues (both in OAC and OSCC) (86). CDK6 is a key regulator of the cell cycle. Induction of YAP1 expression in esophageal cancer cells up-regulated CDK6 expression, increased transcription, and consequently induced the resistance against radiotherapy (86). By blocking YAP1 and CDK6 with the YAP1 inhibitor CA3, and the CDK6 inhibitor LEE001 significantly suppressed esophageal cancer cell growth and CSC properties, particularly in radiation-resistant cells in both OAC and OSCC (86). The combination of LEE001 and CA3 exhibited the highest anti-tumor effects in radiation-resistant cells overexpressing YAP1 and CDK6 in both *in vitro* and *in vivo* by sensitizing resistant tumors to irradiation (86). Thus, it was implied that crosstalk between YAP1 and CDK6 seems to play a pivotal role in conferring radiation resistance and targeting both YAP1 and CDK6 could be a useful therapeutic strategy to treat both esophageal adenocarcinoma and squamous cell carcinoma.

## Targeting Other Pathways in Esophageal Cancer Stem Cells

The pathways discussed above may act alone or in crosstalk with other pathways to induce stem cell properties in cancer cells or can even participate in driving therapy resistance upon interacting with other pathways (51). For example, the mTOR pathway is often activated in cancers and may generate therapy resistance followed by Hedgehog pathway inhibition (87, 88). The mTOR pathway along with Hedgehog and other pathways are associated with the maintenance of CSC phenotypes (89–93). Thus, interrupting mTOR with novel therapeutic could induce a reduction of stemness of cancer cells and sensitize them to the therapies. Metformin, an anti-diabetic agent, for instance, was found to significantly inhibit cell growth in both OSCC and OAC cells and sensitized them to 5-FU by targeting the mTOR signaling pathway in CSCs (80, 87–91). It increased the effectiveness of 5-FU against both OSCC and OAC cells and inhibited their growth *in vitro* and in a xenograft nude mouse model (51). Significant downregulation of mTOR pathway components including phospho-AKT, phospho-S6, phospho-70S6 was seen followed by metformin treatment, which are crucial to maintaining tumor cells’ growth. Furthermore, metformin treatment strongly decreased the expression of stem cell markers such as *Jagged1*, *Shh*, and *YAP1* (51). Therefore, metformin-induced cell growth inhibition *in vitro* and *in vivo* in both OSCC and OAC cells by its ability to reduce the CSCs population as well as inhibition of the mTOR pathway. Furthermore, the synergistic effect of metformin with 5-FU was particularly of interest, because it would potentially provide an opportunity to treat both the CSCs and proliferating cell component at the same time, to effectively increase the sensitivity of chemo-radiation in patients with OSCC and OAC.

The JAK/STAT signaling pathway has been implicated in various physiological processes, and inhibition of this pathway could impede cancer cell growth and induce apoptosis in various cancers (94–96). Cyclooxygenase-2 (COX-2) together with JAK/STAT signaling has been found to be involved tumorigenesis. Specifically, the tumorigenesis pathway is associated with COX-2 upregulation (97, 98). Inhibition of COX-2 with nimesulide, a selective COX-2 inhibitor, results in suppression of the JAK/STAT signaling pathway, which subsequently inhibits the growth of Eca-109 human OSCC cells (52). Nimesulide induced apoptosis in Eca-109 cells by decreasing the expression of COX-2 and survivin and increasing caspase-3 expression (98). Also, nimesulide inhibited the JAK/STAT pathway by downregulating the phosphorylation of JAK2 and STAT3 (52). Inhibition of *in vivo* tumor growth of Eca-109 in xenotransplanted animals followed by a reduction inexpression of p-JAK2 and p-STAT3 were noted in Nimesulide treatment (52). Though Nimesulide could be used to inhibit JAK/STAT signaling pathway in OSCC cells, its effects on CSCs is yet to be evaluated. Thus, further studies are warranted to explore the effect of inhibition of JAK/STAT pathway in CSCs in esophageal cancers.

Epidermal growth factor receptors (EGFRs), a family of receptor kinases, are expressed in various cancers and contribute to a complex signaling cascade, which in turn controls growth, differentiation, adhesion, migration and survival of CSC and non-CSC cancer cells (53, 99). The wide range of roles for EGFRs in cancer progression makes them an attractive candidate for anti-cancer therapy. EGFRs are overexpressed in OSCC and play pivotal roles in the generation of stem-like cells *via* TGF-β (53). They induce EMT in CD44 overexpressing CSC cells derived from OSCC cells (53). CSCs (CD44+/CD24-) were significantly enriched in EPC2T and OKF6T cells (transformed keratinocyte cell lines) overexpressing EGFR, which could induce EMT by TGF-β1 in CSCs derived fromEPC2T and OKF6T cells (53). Interestingly, Erlotinib and Cetuximab (two EGFR inhibitors) significantly inhibited the enrichment of CSCs *via* inhibition of TGF-β1 mediated EMT (**Table 1**). Also, treatment with EGFR inhibitors resulted in increased expression of CD24 in the non-CSC population (CD44-/CD24+cells), indicating that EGFR inhibition could prompt differentiation in non-CSC populations as CD24 is a marker of keratinocyte differentiation (53). These results suggest that inhibition of EGFR may halt EMT by instigating differentiation in non-CSC populations, thereby suppressing enrichment of CSCs *via* inhibition of EMT. However, these EGFR inhibitors do not affect pre-existing CSCs. By contrast, some EGFR inhibitors suppress Zinc finger E-box binding proteins (ZEBs) and induce differentiation of CSCs in OSCC (53). These findings suggested that EGFR inhibition might suppress the expression of ZEBs and induce differentiation in a wider variety of cancers, thereby blocking EMT-mediated enrichment of CSCs.

NF-kβ, another prominent pathway, regulates various biological processes including apoptosis, proliferation, immune response, cell invasion, and cancer stem‐like cells (CSCs) (100). The key proteins in the NF‐κB pathway (e.g., p50, p52, and Rel) were overexpressed in patients with OSCC (101). In addition, the aberrant activation of the NF‐κB signaling pathway is a significant predictor for prognosis and recurrence of OSCC, which makes it a potential target in the treatment of patients with OSCC (102). A natural quinonemethide triterpenoid compound has been isolated from traditional Chinese herbals known as pristimerin, potently inhibited the growth of OSCC xenograft in nude mice (**Table 1**) (54). Pristimerin demonstrated its anti‐OSCC effects through the inhibition of NF‐κB pathway by suppressing tumor necrosis factor α (TNFα)‐induced Iκ Bα phosphorylation, p65 translocation, and the expression of NF‐κB‐ dependent genes (e.g., *p50*, *p52*, and *Rel*).Furthermore, pristimerin inhibited cell proliferation, migration, invasion, induced apoptosis, and eliminated cancer stem-like cells (CSCs) derived from OSCC cells (54). In addition, pristimerin exhibited a synergistic effect on OSCC when combined with 5‐FU (54). These results imply that pristimerin could increase chemo-sensitivity by suppressing the therapy-resistant CSC cell population in OSCCs.

## Targeting MicroRNA Expression in Esophageal Cancer Stem Cells

MicroRNAs (miRNAs/miRs) are a class of small noncoding RNAs approximately 19–25 nucleotides in length, which regulate post-transcriptional gene expression by binding with their target mRNA transcripts (103, 104). Depending on the roles of their target genes, miRNAs can act either as tumor suppressors or oncogenes (105, 106). They are strongly involved in the formation of CSCs by regulating post transcriptional gene expressions in various cancer types (107). Altered expression of particular cancer-associated miRNAs causes significant changes in the level of potential oncogenic and anti-oncogenic proteins, which suggests miRNAs as useful therapeutic targets in cancer (108). Thus, miRNA mediated changes in gene expression in cancer has become a subject undergoing intense research nowadays.

**Figure 2:**
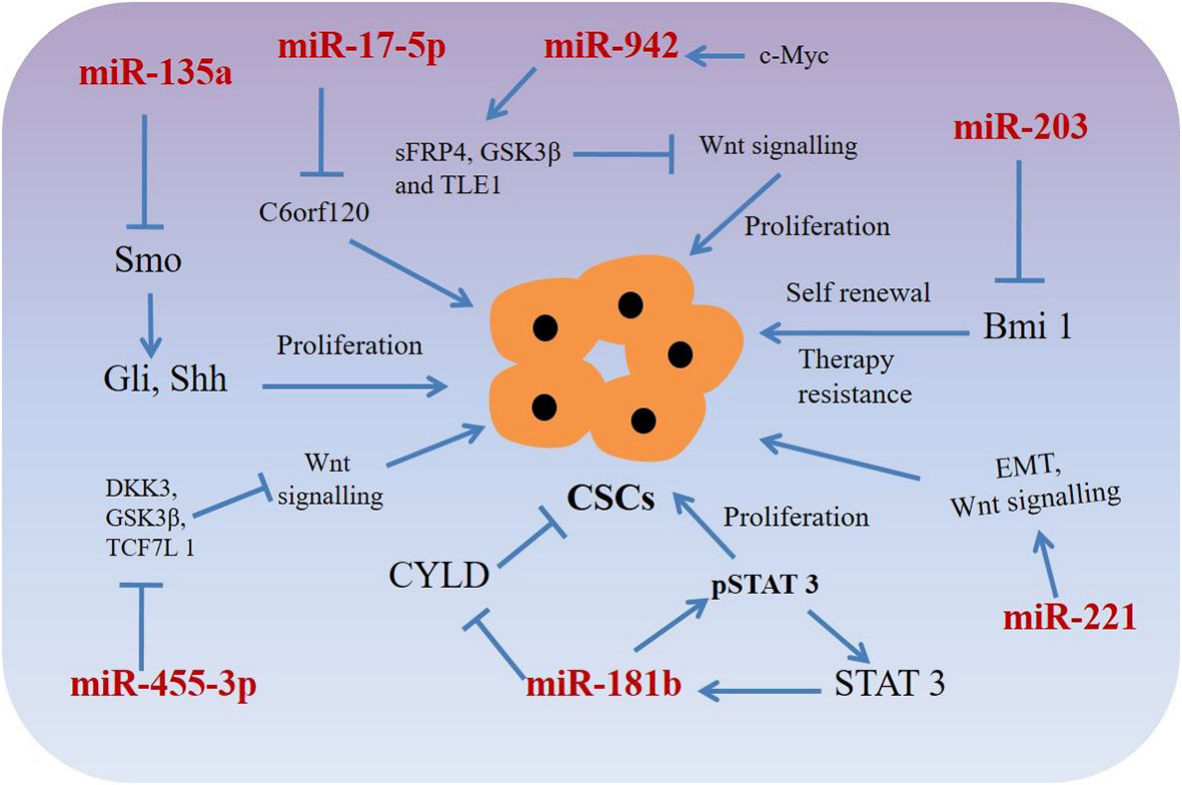
miRNAs targeting phenotypic markers in esophageal cancer stem cells (CSCs). miRNAs upregulate or downregulate the genes related to proliferation, sphere-formation, and therapy resistance.

MicroRNAs could act as molecular markers of cancer stem-like cells in esophageal cancer. Thereby, novel therapeutic strategies targeting miRNAs in CSCs have the potential to eradicate CSCs population, resulting in the improved clinical outcomes for patients with esophageal squamous cell carcinoma or adenocarcinoma (**Table 2**, **Figure 2**) (109–111, 116–118). For example, miRNA-203 is downregulated in cancer stem-like cells (Side population generated from OSCC (EC9706) cells) and expression of miR-203 was inversely associated with the expression of stem cell self-renewal factor Bmi-1 (109). Comparison of expression ofBmi-1 between SP and non-SP cells revealed that Bmi-1 was highly expressed in SP cells and its expression was significantly diminished during the differentiation from SP to non-SP cells (109, 110, 118). Therefore, miR-203 and Bmi-1 appear to play important roles in the generation of cancer stem-like cells in OSCC. In addition, lentiviral mediated expression of miR-203 resulted in decreased colony formation ability of SP cells, which was associated with the resistance to chemotherapy and responsible for tumorigenesis in nude mice (109). Since miR-203 and Bmi-1 were inversely expressed in SP cells, Bmi-1 might be a direct target of miR-203, thus therapeutics targeting miR-203 or Bmi-1could have the potential to eradicate CSCs in OSCC.

**Table 2:** MicroRNAs associated with functions of esophageal cancer stem cells.

MicroRNAs	Expression pattern	Carcinoma (s)	Function	Reference
miRNA-203	Downregulated	OSCC	Expression of miR-203 results in decreased colony formation ability of SP cells by downregulating the expression of Bmi1	(109)
miR-181b	Upregulated	OSCC	miR-181b binds with 3′-untranslated region (UTR) of CYLD mRNA to positively regulate the stemness of esophageal cancer cells miR-181b together with STAT3 regulate stemness of esophageal cancer cells by maintaining feedback loop *via* CYLD pathway	(110)
miR-135a	Downregulated	OSCC	Overexpression of miR-135a decreases the expression of Gli1, Gli2, and Shh, which as a result reduces the proliferation, migration, and invasion of cancer cells and promotes apoptosis	(111)
miR-942	Up-regulated	OSCC	Upregulation of mir-942 promotes cancer stem cell-like traits and tumor sphere formation in OSCC	(112)
miR-455-3p	Up-regulated	OSCC	Promotes chemoresistance and tumorigenesis of OSCC cells	(113)
miR-17	Down-regulated	OAC	Expression of miR-17-5p significantly sensitizes radioresistant cells to X-ray radiation and enhanced the repression of genes such as *C6orf120*	(114)
miR-221	Up-regulated	OAC	Knockdown of miR-221 in 5-flurouracil resistant cells decreases cell proliferation, increases apoptosis, restores chemosensitivity, and leads to inactivation of the stem cell pathway Wnt/β-catenin by activation of DKK2 activity	(115)

OSCC, esophageal squamous cell carcinoma; OAC, esophageal adenocarcinoma.

Another miRNA, miR-181b in association with STAT3, plays a significant role in stem cell properties of esophageal squamous cell carcinoma stem-like cells (110). Isolating sphere-forming cells from OSCC cells (Eca109) exhibited proliferation and tumorigenicity characteristics of CSCs in a mouse xenograft model (110). The sphere-forming cells demonstrated cancer stem-like cell properties such as an enhanced population of CD44+/CD24- cells, increased stemness factors, mesenchymal marker expression, ATP-binding cassette (ABC) transporters and tumorigenicity *in vivo* when compared to that of parental cells (110). A mutual regulation between the signal transducer and activator transcription 3 (STAT3, a transcription factor) and miR-181b controls the sphere-forming cells’ proliferation and apoptosis resistance in esophageal cancer stem-like cells. STAT3 directly activated miR-181b transcription in a sphere-forming cells, which in turn potentiated p-STAT3 activity (110). Mechanistically, miR-181b binds with 3′-untranslated region (UTR) of cylindromatosis (CYLD) mRNA and regulates CYLD expression, which in turn regulates sphere-forming cells *via* modulating the STAT3/miR-181b loop in esophageal CSCs.

MicroRNAs such asmiR-135a may regulate biological behaviors of CSCs in OSCC through the Hedgehog signaling pathway by targeting its component SMO (111). Expressions of hedgehog pathway proteins such as SMO, Gli1, Shh, and Gli2 were happened to be increased and the expression of miR-135a was decreased in in esophageal CSCs of squamous cell carcinoma. However, exogenous overexpression of miR-135a or silencing of SMO decreased the expression of Gli1, Gli2, and Shh, resulting in reduced proliferation migration, invasion and increased apoptosis of CSCs derived from esophageal cancer cells (111). Interestingly, silencing of miR-135a was associated with increased carcinogenic capability of miR-135a in CSCs derived from OSCC (111). These results suggest that miR-135a mediated inhibition of CSCs derived from esophageal squamous cell carcinoma cells through suppression of the SMO/Hedgehog axis may act as a potential therapeutic option for patients with the carcinoma.

Another example of a miRNA promoting stem cell-like characteristics is miR-942, which in OSCCs causes activation of the Wnt/β-catenin signaling pathway (112). miR-942 was significantly upregulated in OSCC and was correlated with poor prognosis in patients with OSCC. Upregulation of miR-942 promoted cancer stem-like cell (CD90+ cells) traits in OSCC, whereas inhibition of miR-942 decreased tumor sphere formation and inhibited the expression of pluripotency-associated markers in the stem-like cells (112). Moreover, *in vivo* assays demonstrated thatmiR-942 overexpressing cells form larger tumors and display higher tumorigenesis capacity (112). miR-942 upregulates the Wnt/β-catenin signaling activity *via* directly targeting FRP4, GSK3β, and TLE1, which are prominent negative regulators of the Wnt/β-catenin signaling cascade (112). In addition, c-myc (a stem cell pluripotency-associated marker) directly binds to the miR-942 promoter and increased its expression, resulting in increased CSC mediated tumorigenesis (112). Considering the oncogenic role of miR-942 in OSCC, miR-942 might be an attractive therapeutic target for patients with OSCC.

Also, dysregulation of miR-455-3ppromoted chemoresistance and tumorigenesis of OSCC cells (113). Interestingly, treatment with a miR-455-3p antagomir significantly chemo-sensitized OSCC cells and decreased CD90+ and CD271 + cell populations (a CSC phenotype) through inhibition of various stemness-associated pathways including Wnt/β-catenin and TGF- β signaling (113). miR-455-3p targets several negative regulators e.g. DKK3, GSK3β, TCF7L 1, IGFBP4 etc. (Wnt/β-catenin pathway components) and Smurf2, NEDD4L, FKBP1A, BAMB I, etc. (TGF-β/Smad pathway components), resulting in inactivation of Wnt/β-catenin and TGF-β signaling in CSCs derived from OSCC cells (113). Association of miR-455-3p levels with chemoresistance and overall/relapse-free survival of patients with OSCC, indicating miR-455-3p antagonist could have potential as effective therapeutics for patients with OSCC. Another miRNA, miR-17 associated with the radio-resistant property of OAC cancer stem-like cells (114). An *in vitro* isogenic model using radio-resistantOE33 R cells derived from OE33 OAC cells demonstrated increased expression of CSC-associated markers and had enhanced tumorigenicity *in vivo* and increased holoclone forming capacity (114). Also, radio resistantOE33 R cells have increased ALDH activity. However, an *in vitro* study suggested that exogenous expression of miR-17-5p significantly sensitized radio-resistant cells to radiation therapy by repression of *chromosome 6 open reading frame 120* (*C6orf120)* expression (114). This study sheds novel insights into the role of miR-17-5p as a potential prognostic biomarker in patients with esophageal adenocarcinomas.

Additionally, miR-221 is another miRNA upregulated in 5-FU resistant esophageal cancer cells (OAC) as well as in human OAC tissues (115). DKK2, a putative inhibitor of Wnt signaling was identified as a potential target for miR-221. Importantly, miR-221 knockdown in 5-FU resistant cells resulted in decreased cell proliferation, increased apoptosis, restored chemo-sensitivity, and led to inactivation of the stem cell pathway Wnt/β-catenin by activation of DKK2 activity (115). In addition, reduction of miR-221 expression resulted in alteration of EMT-associated genes e.g. *E-cadherin* and *vimentin* and slowed xenograft tumor growth in nude mice (115). Furthermore, a substantial dysregulation of Wnt/β-catenin signaling and chemoresistance target genes such as *CDH1*, *CD44*, *MYC*, and *ABCG2* was reported as a result of miR-221 modulation in OAC (115). miR-221 may, therefore, could act as a prognostic marker and therapeutic target for patients with OAC.

## Targeting Hypoxia-Related Pathways in Esophageal Cancer to Eliminate Cancer Stem Cells

**Figure 3:**
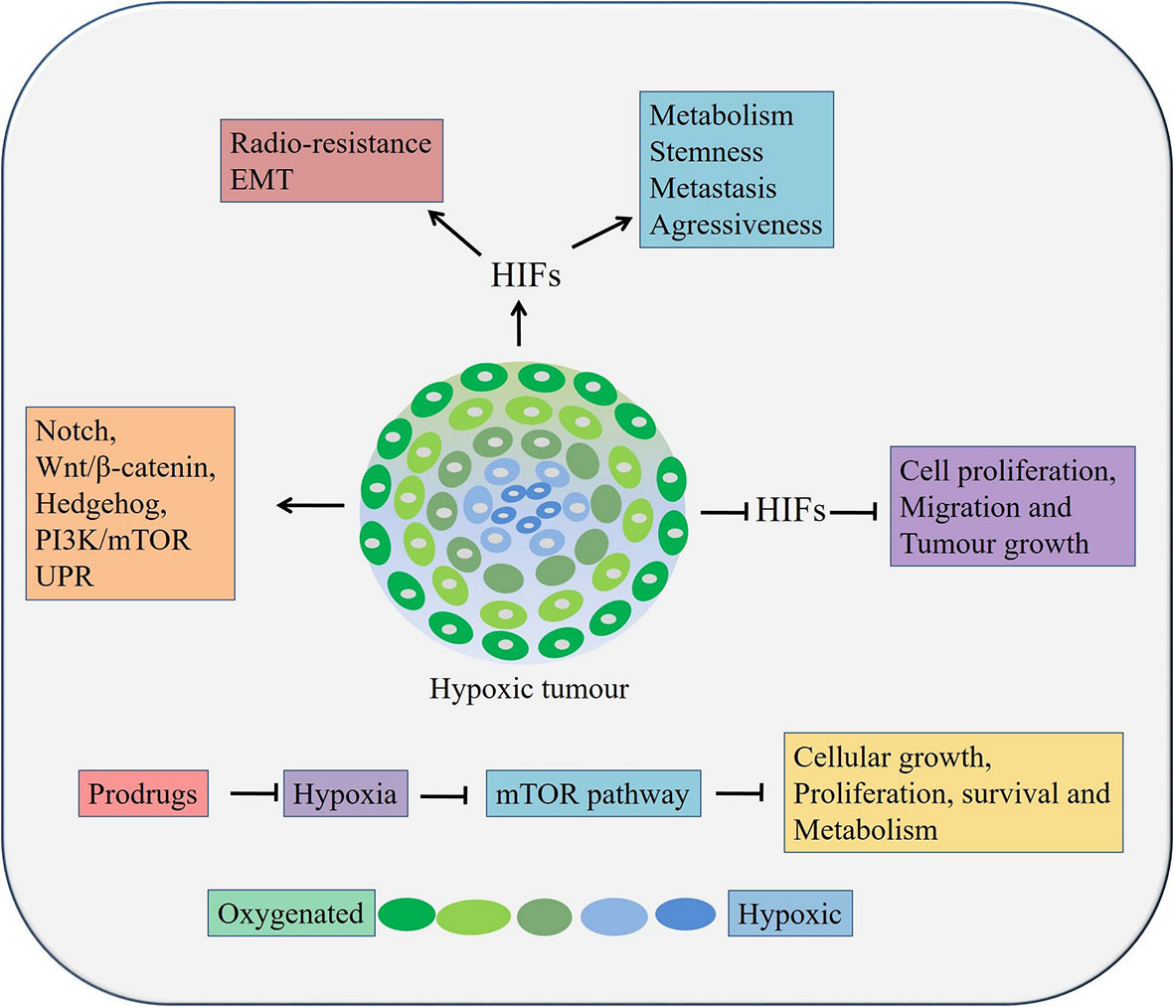
Role of hypoxia in esophageal cancers. Hypoxia can lead to cancer cell growth, metastasis, stemness, and therapy resistance through aberrant activation of pathways, inducing EMT processes etc. HIFs, Hypoxia inducible factors.

Hypoxia, resulting from low oxygen concentration and nutrition deprivation, is a very common scenario in locally advanced solid tumors (119, 120). It regulates hypoxia-inducible factor (HIF) 1 and 2, which in turn can play critical roles in cancer metabolism, stem cell proliferation, maintaining aggressiveness and metastatic potential of both OSCC and OAC cells (**Figure 3**) (119, 120). Overexpression of HIFs also reduces radio-sensitivity (121, 122) and induces EMT in cancer cells (123, 124). On the other hand, inhibition of HIF1α resulted in suppression of tumorigenicity of OSCC cells in both *in vitro* and *in vivo* (125). At tissue levels, hypoxia and HIF1α are associated with therapy resistance and poor prognosis in patients with OSCC and OAC (126–129). Moreover, hypoxia regulates EMT and cancer stemness in various cancers by targeting Notch, Wnt/β-catenin, Hedgehog, PI3K/mTOR and unfolded protein response (UPR) pathways (130).

In esophageal cancer, a lower level of oxygen increases the CSC population, suggesting the need to target hypoxia in order to eradicate all tumor cells, especially the CSC population (131). It was reported that the expression of HIF‑1α and CSC‑related genes conditions were upregulated under hypoxic condition. A significant reduction of cell proliferation, migration and tumor growth was occurred followed by HIF‑1α knockdown in OSCC cells *in vivo* (131). In addition, knockdown of HIF‑1α also inhibited spheroid formation, inhibited expression of CSC‑related genes and Wnt/β‑catenin target genes, thereby decreased Wnt/β‑catenin activity CSCs derived from OSCC (131). Therefore, targeting hypoxia or its related factor and at the same time, inhibiting Wnt/β‑catenin might be an attractive option against patients with both OSCC and OAC. There are two main strategies targeting tumor hypoxia. Firstly, by applying bio-reductive prodrugs and secondly, inhibiting molecular targets associated with hypoxia using molecular inhibitors (132). A few prodrugs, for example, Tirapazamine, Apaziquone, TH-302, PR-104, Banoxantrone, and RH1 are effective in other solid cancers and are in clinical trials in minimizing tumor hypoxia (132). These prodrugs could be utilized against hypoxia in esophageal cancers. Interestingly, inhibition of the PI3K/mTOR pathway or a hypoxia may lead to activation of autophagy and could be used as an alternative therapeutic modality in esophageal cancers (130). The mTOR pathway negatively regulates autophagy in hypoxic conditions along with regulating cellular growth, proliferation, survival and metabolism (133). Thus, targeting the mTOR pathway mediated autophagy by Bafilomycin and Chloroquine could be useful against CSCs in both OAC and OSCC (73).

Finally, clinical trials targeting esophageal CSCs registered at https://clinicaltrials.gov/ were examined. To the best of our knowledge there is only a study using Fursultiamine, a nutrition supplement is undergoing a phase II clinical trial against OSCC patients in Taiwan in combination with concurrent chemo-radiation therapy to target CSCs (NCT02423811). Fursultiamine suppress OCT-4, SOX-2, NANOG expression and decreased ABCB1 and ABCG2 in tumor spheres. These findings encouraged the researchers to undertake a phase II trial to identify the effect of Fursultiamine combined with concurrent chemo-radiation therapy in ESCC patients. The outcome of the trial is not reported yet, however, they suggested that stem cell markers in clinical specimens collected before and after concurrent chemo-radiation therapy would be evaluated to identify whether Fursultiamine is effective against CSCs or not.

## Concluding Remarks

Current conventional anticancer therapies are unable to eliminate CSCs. Therefore, relapse can occur, and CSCs can enable tumors to develop with further resistance to treatment and with more biological aggressiveness. In esophageal cancer, accumulating information has led to the hypothesis that the CSC population could be the seeds of carcinogenesis and are associated with therapy resistance and cancer recurrence. Thus, targeted therapy against CSCs could offer new options approaches to eliminate the malignant phenotypes of cancer without causing any harm to normal stem cells. In addition, careful analysis of a patient’s specific tumor may lead to a personalized approach, where both CSCs and the bulk tumor can potentially be eradicated. Eradicating both CSCs and bulk tumor should lead to a more promising outcome for patients with esophageal cancers. In some patients, conventional chemotherapy, surgical strategy along with targeted therapy will ultimately provide a more durable cure to this disease.

## Author Contributions

PD drafted the manuscript. FI synthesized the concept and edited the manuscript. AL supervised the project and edited the manuscript. RS edited the concept and did the English proofreading. All authors contributed to the article and approved the submitted version.

## Conflict of Interest

The authors declare that the research was conducted in the absence of any commercial or financial relationships that could be construed as a potential conflict of interest.
